# β-Catenin/LEF-1 transcription complex is responsible for the transcriptional activation of LINC01278

**DOI:** 10.1186/s12935-021-02082-9

**Published:** 2021-07-17

**Authors:** Shaojian Lin, Weiwei Zhang, Ziwen Shi, Langping Tan, Yue Zhu, Honghao Li, Xinzhi Peng

**Affiliations:** 1 Department of Thyroid Surgery, Sun Yat-Sen Memorial Hospital, No. 107 of Yanjiangxi Road, Guangzhou, 510120 Guangdong People’s Republic of China; 2 Sun Yat-Sen University, No. 74 of Zhongshaner Road, Guangzhou, 510080 Guangdong People’s Republic of China; 3 Department of Ultrasound, Guangdong Province Traditional Chinese Medical Hospital, No. 111 of Dade Road, Guangzhou, Guangdong People’s Republic of China

**Keywords:** LINC01278, LEF-1, β-Catenin, Wnt/β-catenin signaling pathway, Papillary thyroid carcinoma

## Abstract

**Background:**

Our previous study shows that LINC01278 inhibits the malignant proliferation and invasion of papillary thyroid carcinoma (PTC) cells by regulating the miR-376c-3p/DNM3 axis. However, the regulation mechanism of LINC01278 expression in PTC cells is still unclear.

**Methods:**

The luciferase reporter and ChIP assays were used to confirm the binding of LEF-1 to the putative promoter site of *LINC01278* gene. The RNA immunoprecipitation and RNA pulldown were used to determine the enrichment of LINC01278 in β-catenin protein. The proteasome inhibitors (MG132) was used for detecting the β-catenin ubiquitination-proteasome degradation. Wnt/β-catenin specific agonists (LiCI), inhibitors (WiKI4) and TOP/FOP-flash reporter assay were used for detecting the activation of Wnt/β-catenin signal. Western blot was used to detected the expression of target proteins.

**Results:**

The online PROMO algorithm determines a putative LEF-1 binding site on LINC01278 promoter, the LEF-1 binds to the putative promoter site of *LINC01278* gene, and β-catenin enhances the binding of LEF-1 to the *LINC01278* gene promoter. Furthermore, LINC01278 negatively regulated the protein accumulation of β-catenin in the cytoplasm, into nucleus, and ultimately inhibited the transcription of downstream target genes activated by Wnt/β-catenin signal. The results of RNA immunoprecipitation and RNA pulldown proved the direct binding of LINC01278 to β-catenin protein. In addition, the combination of LINC01278 and β-catenin promotes the β-catenin ubiquitination-proteasome degradation.

**Conclusion:**

In summary, we found the transcriptional activation of LINC01278 by the β-catenin/LEF-1 transcription factor, and the negative feedback regulation of LINC01278 onβ-catenin signal.

## Background

Thyroid cancer is the most common malignant tumor of the endocrine system [1, 2]. In the past 20 years, its incidence has increased rapidly worldwide [3]. Although it is unclear whether the incidence increase reflects an increase in the true incidence or an improvement of diagnostic technology [4, 5]. Papillary thyroid carcinoma (PTC) is the most common subtype of thyroid malignanncies, accounting for more than 80% of thyroid cancer, which originates from thyroid follicular epithelial cells [6]. A large proportion of thyroid cancers, especially PTCs, usually exhibit indolent biological behavior and have a good prognosis [7]. However, some patients show aggressive clinical manifestations, such as distant metastasis and recurrence [8]. In the case of high morbidity and low mortality, the pathogenesis of PTC should be further studied.

Long-chain no-coding RNAs (lncRNAs) are transcripts that are more than 200 bp in length and lack protein coding ability [9]. Numerous studies have shown that lncRNAs play a vital role in the occurrence and progression of cancer [10]. Our previous study showed that LINC01278 was significantly down-regulated in PTC tissues and cell lines [11]. The low expression of LINC01278 was related to tumor size, lymph node metastasis, pathological grade and clinical stage. In addition, LINC01278 inhibited the malignant proliferation and invasion of PTC cells by regulating the miR-376c-3p/DNM3 axis. However, the regulation mechanism of LINC01278 expression in PTC cells is still unclear.

Typical Wnt signal transduction is conservative and plays a key role in normal development and cancer progression. In the absence of Wnt ligands, the central protein β-catenin binds to the GSK3β/Axin/APC complex. The latter phosphorylates β-catenin, and the phosphorylated β-catenin is degraded by the ubiquitination-proteasomal system [12]. In the presence of a Wnt ligand, the Wnt ligand binds to the Wnt receptor (Frizzled and LRP5/6) and subsequently activates the Dsh/Dvl protein. The Dsh/Dvl protein interacts with the GSK3β/Axin/APC complex, causing the release of β-catenin. β-catenin accumulates in the cytoplasm and enters the nucleus. After entering the nucleus, β-catenin binds to TCF/LEF-1 transcriptional regulators to activate the transcription of target genes [13].

In this study, we will explore the transcriptional regulation of the β-catenin/LEF-1 transcription complex on the *LINC01278* gene, and the negative feedback mechanism of LINC01278 on the post-transcriptional regulation of β-catenin.

## Methods

### Cell culture

Human thyroid cancer cell lines (TPC-1 and BCPAP) were obtained from ATCC (American Type Culture Collection) (Manassas, VA, USA), and cultured in DMEM (Dulbecco’s modified Eagle’s medium) (Invitrogen, Carlsbad, CA, USA) supplemented with 10% fetal bovine serum (FBS) (Invitrogen), 100 U/mL penicillin and 100 μg/mL streptomycin (Hyclone, South Logan, UT, USA), and incubated at 37 °C in a humidified atmosphere containing 5% CO_2_.

### Cell transfection and treatment

LEF-1, β-catenin and LINC01278 overexpression vectors were bought from Kang-cheng Biotechnology Co (Guangzhou, China). Small interfering RNA (siRNA) against LEF-1, β-catenin and LINC01278 were designed and purchased from RiboBio Co., Ltd. (Guangzhou, China). All vectors and siRNAs were transfected into TPC-1 and BCPAP cells using Lipofectamine 2000 (Invitrogen) following the manufacturer’s instructions. The Wnt/β-catenin specific agonists (LiCI), inhibitors (WiKI4) and proteasome inhibitor MG132 were purchased from Sigma (Beijing, China).

### Analysis of online database

The online PROMO algorithm analysis (http://alggen.lsi.upc.es/cgi-bin/promo_v3/promo/promoinit.cgi?dirDB=TF_8.3) was used to predict the binding of transcription factors to the LINC01278 promoter sequence [14, 15].

### Luciferase reporter assay

The wild promoter sequence of LINC01278 (WT) and mutant promoter sequence of LINC01278 (MUT) in which the LEF-1-binding site was mutated were cloned into a pGL3-Basic vector (Promega), respectively. The recombinant pGL3-Basic vector was co-transfected into TPC-1 and BCPAP cells with LEF-1 overexpression (LEF-1) or negative control (NC) plasmid. After 48 h, the cells were lysed, and the luciferase activity was analyzed using Dual-Luciferase Reporter Kit (Promega) and Varioskan Lux detection system (Thermo Scientific), which was standardized to Renilla.

### Chromatin immunoprecipitation (ChIP) assay

Cells were fixed and crosslinked in 1% formaldehyde for 10 min at 37 °C and incubated with protease inhibitors. Chromatin was isolated and enzymatically fragmented using an EZ-Zyme Chromatin Prep Kit (17,375, Merck). Rabbit anti-LEF-1 antibody (EPR2029Y, 1:50, Abcam) or nonspecific IgG (1:200, Sigma) was used to precipitate DNA crosslinked with the LEF-1. The immunoprecipitated promoter fragment containing the LEF-1 response element was probed by PCR using primers targeting the regulatory region of *LINC01278* gene and visualized by agarose gel electrophoresis.

### Western blot

Cells were lysed using RIPA buffer. The lysate was separated by 10−15% SDS–polyacrylamide gel electrophoresis (SDS-PAGE) and transferred to a polyvinylidene difluoride (PVDF) membrane (Millipore, Billerica, MA). The membrane was blocked with 5% nonfat milk and incubated with anti-LEF-1 (1:1000, ab137827, Abcam), anti-β-catenin (ab16051, 1:1000, Abcam), anti-CCND2(ab230883, 1:1000, Abcam), anti-CyclinD1 (ab226977, 1:1000, Abcam), anti-MYC (ab32072, 1:1500, Abcam), anti-SOX4 (ab86809, 1:1000, Abcam), anti-Ubiquitin (ab134953, 1:1000, Abcam) and anti-GAPDH (ab8245, 1:1000, Abcam) antibodies overnight at 4 °C. Then the membrane was washed with TBST three times and incubated with the secondary antibody (Abcam, Cambridge, MA) for 1.5 h at room temperature. The ECL chromogenic solution was used to display the chemiluminescence of the bands. The Quantity One 4.4.0 software was used for densitometry determination.

### Quantitative real-time PCR

Total cellular RNAs were isolated and purified using TRIzol reagents (Invitrogen) and cDNA synthesis was performed using PrimeScript RT reagent Kit (TaKaRa, Otsu, Shiga, Japan) according to the manufacturer’s instructions. Real-time PCR was performed using SYBR Premix Ex Taq TM (TaKaRa) in the ABI 7900HT Fast Real-time PCR system (Applied Biosystems, Foster City, CA, USA). The comparative cycle threshold (CT) (2-^C^T) method was used to measure the LINC01278 level, and GAPDH was used as an internal control. The sequence of primers was as follows: LINC01278, F: 5′-CTGTTGCCCTCCTTCACCTA-3′, R: 5′-TGGTCTACAGGGAGTGCAAG-3′; GAPDH, F: 5′-TGTTCGTCATGGGTGTGAAC-3′, R: 5′-ATGGCATGGACTGTGGTCAT-3′.

### CCK-8 assays

After 48 h of transfection, the old medium was removed and fresh medium containing 10% CCK-8 reagent was added. After incubating for 2 h at 37 °C, the absorbance of each well at 450 nm was detected, and a growth curve was draw.

### Apoptosis detection

1 × 10^5^ transfected cells were seeded into each well of a 6-well plate and cultured in FBS-free medium for 48 h. Then, the cells were digested and resuspended to 1 × 10^6^ cells/ml with binding buffer. 5 μl FITC-Annexin V and 5 μl PI were added. Cell apoptosis was analyzed using FACScan flow cytometry system (BD Biosciences, San Jose, CA) and FlowJo V7 software (Tree Star, Ashland, OR).

### RNA immunoprecipitation (RNA IP)

Cells were fixed and crosslinked in 1% formaldehyde, and lysed using RIPA buffer supplemented with protease-inhibitor cocktail and RNase inhibitor. The cell lysates were incubated with magnetic beads conjugated with anti-β-catenin antibody (Millipore). Mouse IgG (Millipore) was used as the negative control. The immunoprecipitated RNA was extracted from the eluate, and the enrichment of LINC01278 or control ACTB was analyzed by quantitative RT-PCR.

### RNA isolation of nuclear and cytoplasmic fractions

The NE-PER™ Nuclear and Cytoplasmic Extraction Reagents Kit (Thermo Scientific, USA) was applied to isolate and collect cytosolic and nuclear fractions.

### TOP/FOP-flash reporter assays

Cells were seeded in a 96-well plate, and the TOP/FOP-Flash (Genechem) was co-transfected into cells along with LINC01278 siRNA or overexpression plasmid. Luciferase activity was measured using the Promega Dual-Luciferase Reporter Assay System at 24 h after transfection. The luciferase activity was normalized to the Renilla luciferase activity.

### RNA pull-down assay

The purified biotinylated LINC01278 transcripts were purchased from Sangon Biotech (Shanghai, China). 1 mg of cell lysates was incubated with 3 mg of purified biotinylated transcripts for 1 h at 25 °C. The biotin-coupled RNA complexes were isolated by streptavidin agarose beads (Invitrogen), and the protein expression in the pull-down material was detected by western blot.

### Statistical analysis

Statistical analysis was performed using SPSS 22.0 software (SPSS, Armonk, NY, USA). The data came from at least three independent experiments, evaluated by Student’s t test, and presented as mean ± SD. *P* < 0.05 was considered as statistically significant.

## Results

### LEF-1 binds to the promoter region of the LINC01278 gene

**Fig. 1:**
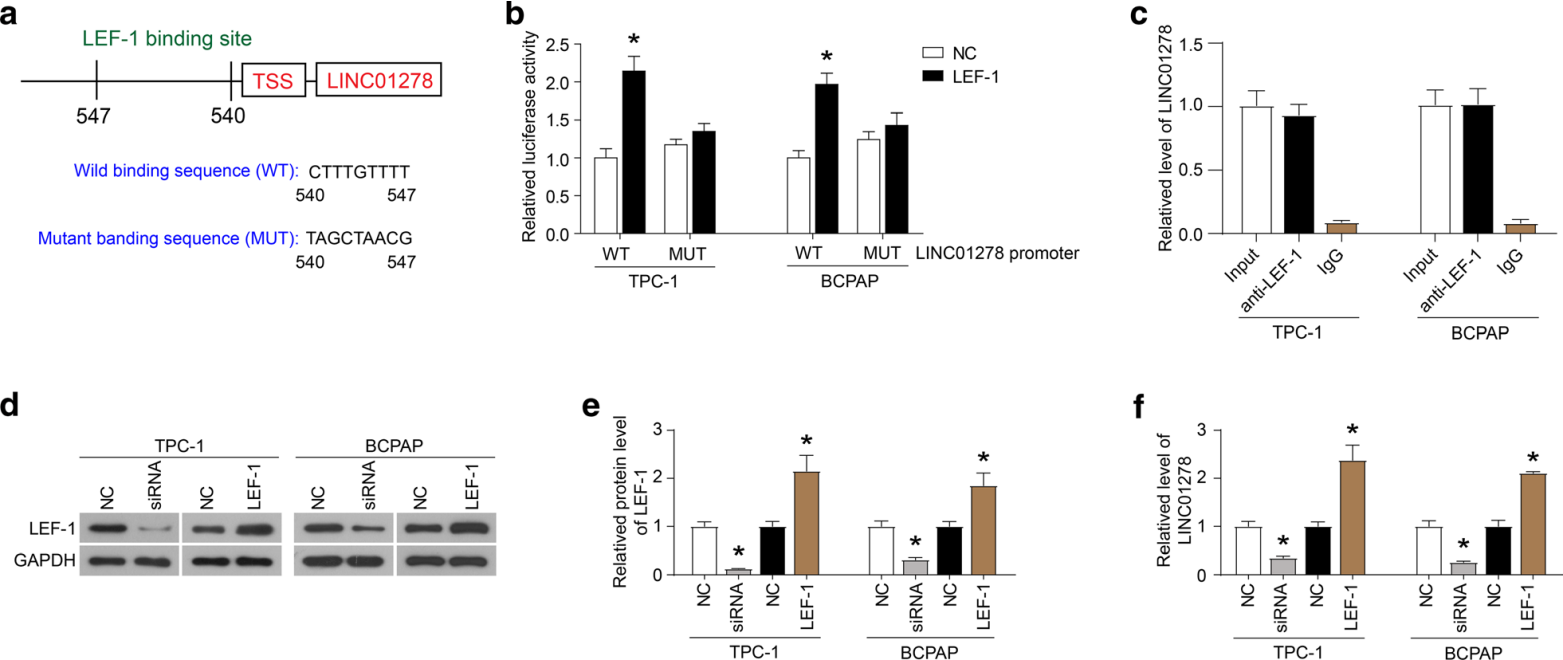
LEF-1 binds to the promoter region of the *LINC01278* gene. **A** The putative LEF-1 binding site on LINC01278 promoter sequence determined by online PROMO algorithm. **B** The luciferase activity of TPC-1 and BCPAP cells which were co-transfected with LINC01278-WT and LEF-1, or LEF-1 and LINC01278-MUT detected by luciferase reporter assays. **C** The immunoprecipitated promoter fragment containing the LEF-1 response element was probed by PCR using primers targeting the regulatory region of *LINC01278* gene. **D** and **E** The protein expression of LEF-1 in TPC-1 and BCPAP cells which were transfected with LEF-1 siRNA (siRNA) or overexpression plasmid (LEF-1). **F** The relative expression of LINC01278 in TPC-1 and BCPAP cells which were transfected with LEF-1 siRNA (siRNA) or overexpression plasmid (LEF-1). *P < 0.05

**Fig. 2:**
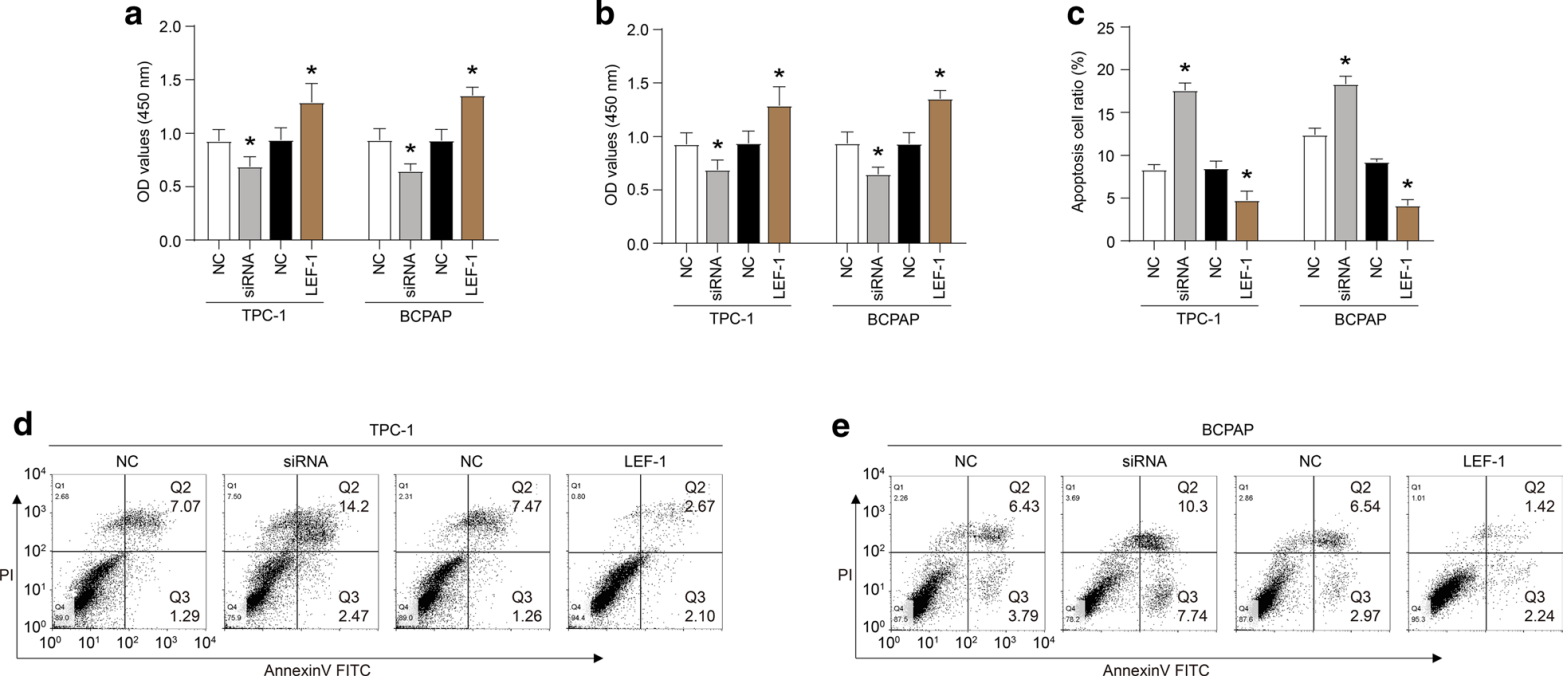
LEF-1 promotes the proliferation of TPC-1 and BCPAP cells. **A** and **B** The proliferation of TPC-1 and BCPAP cells which were transfected with LEF-1 siRNA (siRNA) or overexpression plasmid (LEF-1). **C**, **D** The apoptosis ratio of TPC-1 and BCPAP cells which were transfected with LEF-1 siRNA (siRNA) or overexpression plasmid (LEF-1). *P < 0.05

Firstly, the online PROMO algorithm was used to analyze the promoter sequence of *LINC01278* and determine a putative LEF-1 binding site (Fig. 1A). Then, the luciferase reporter assays further showed that the luciferase activity of the TPC-1 and BCPAP cells co-transfected with LINC01278-WT and LEF-1 was significantly increased compared with the NC group. The luciferase activity of the cells co-transfected with LEF-1 and LINC01278-MUT showed no significant change compared with the NC group (Fig. 1B). ChIP assays further demonstrated the binding of LEF-1 to the *LINC01278* promoter (Fig. 1C). In addition, when LEF-1 was knocked down, LINC01278 levels were significantly reduced (Fig. 1D–F). Moreover, when LEF-1 was over-expressed, the level of LINC01278 increased significantly (Fig. 1D–F). Furthermore, the down-regulation of LEF-1 expression inhibited the viability of TPC-1 and BCPAP cells and induced apoptosis, while the up-regulation of LEF-1 expression promoted the cell viability and inhibited apoptosis (Fig. 2A–E). These data indicated that LEF-1 could bind to the promoter region of *LINC01278* gene to activate its transcription and participate in the regulation of cell viability and apoptosis of TPC-1 and BCPAP cells.

### Overexpression of β-catenin enhances the transcription of LINC01278 activated by LEF-1

**Fig. 3:**
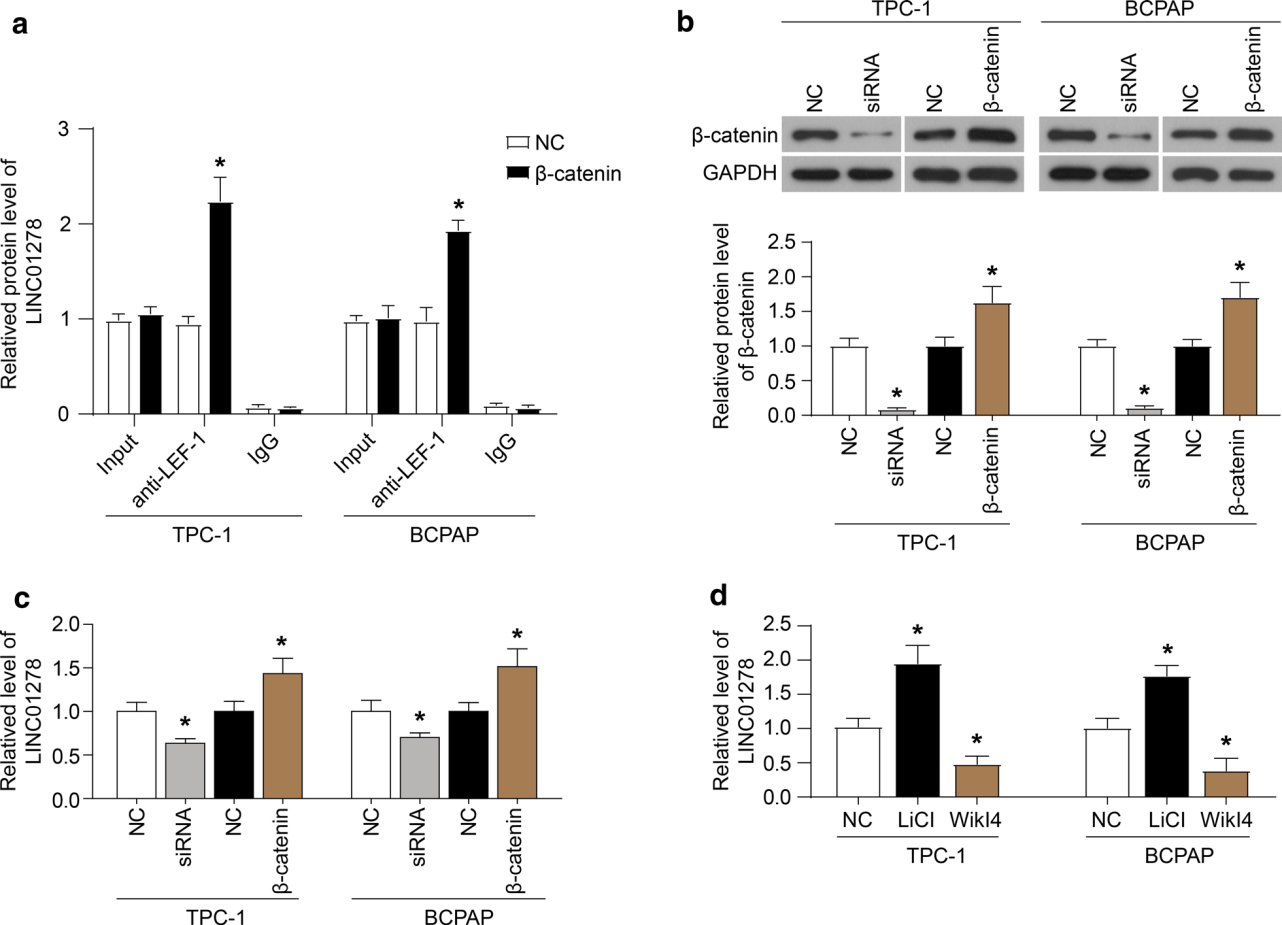
Overexpression of β-catenin enhances the transcription of *LINC01278* activated by LEF-1. **A** The immunoprecipitated promoter fragment containing the LEF-1 response element was probed by PCR using primers targeting the regulatory region of *LINC01278* gene in TPC-1 and BCPAP cells which overexpressed β-catenin. The expression of β-catenin protein (**B**) and LINC01278 (**C**) in TPC-1 and BCPAP cells which were transfected with β-catenin siRNA (siRNA) or overexpression plasmid (β-catenin). **D** The LINC01278 levels in TPC-1 and BCPAP cells which were treated with Wnt/β-catenin specific agonists (LiCI) and inhibitors (WiKI4). *P < 0.05

Based on β-catenin as a cofactor of TCF/LEF-1 transcription activation, we studied the effect of β-catenin on the transcription of LINC01278 activated by LEF-1. The results of ChIP showed that overexpression of β-catenin increased the binding of LEF-1 to the *LINC01278* promoter (Fig. 3A). Furthermore, down-regulation of β-catenin decreased the expression of LINC01278, while the up-regulation of β-catenin increased the expression of LINC01278 (Fig. 3B and C). In addition, Wnt/β-catenin specific agonists (LiCI) and inhibitors (WiKI4) was applied. LiCI significantly increased the intracellular LINC01278 levels, and WiKI4 reduced the intracellular LINC01278 levels (Fig. 3D). These results indicated that β-catenin was involved in the transcription of LINC01278 regulated by LEF-1.

### LINC01278 interacts with β-catenin

**Fig. 4:**
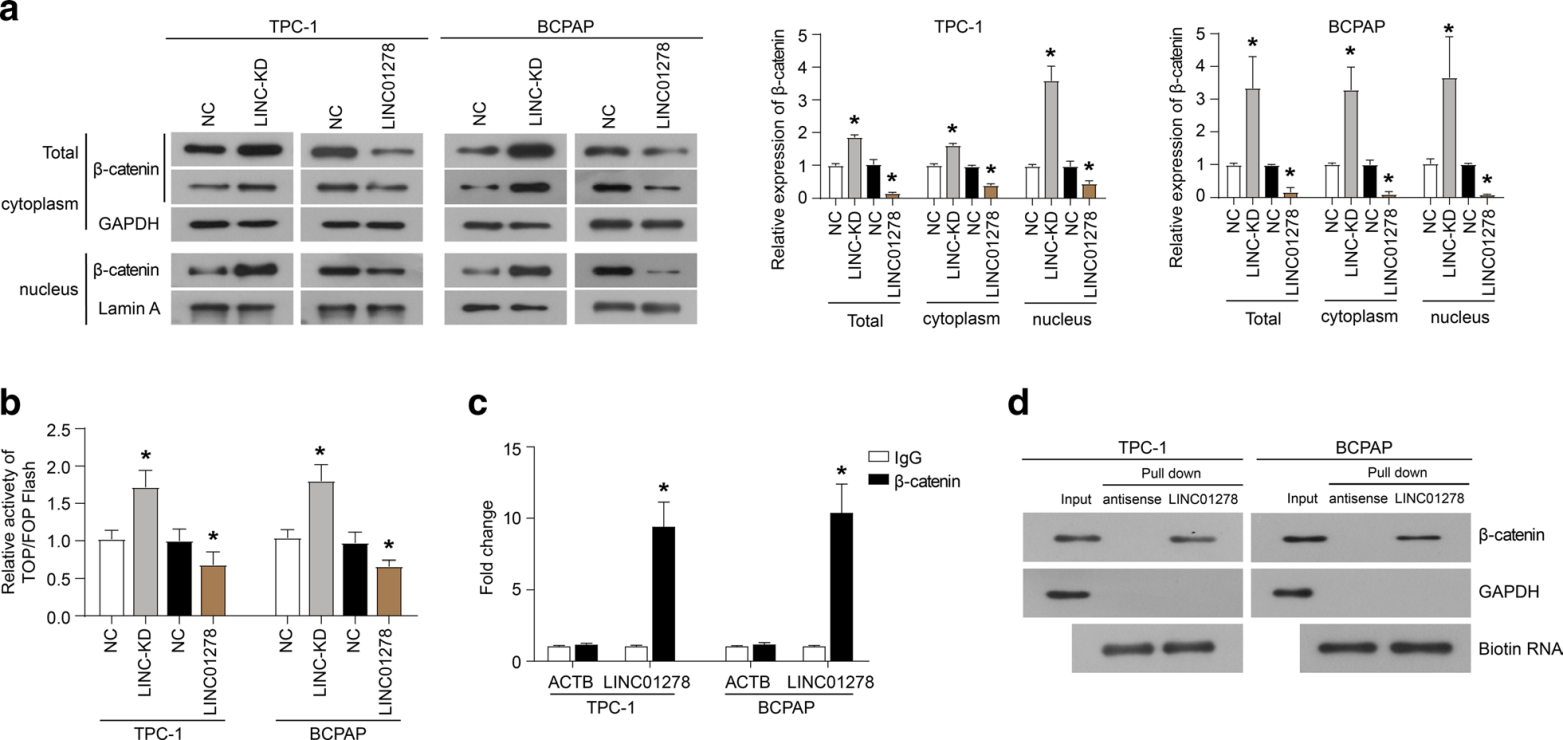
LINC01278 interacts with β-catenin. **A** The protein expression of β-catenin in TPC-1 and BCPAP cells which were transfected with LINC01278 siRNA (LINC-KD) or overexpression plasmid (LINC01278). **B** The luciferase activity of TPC-1 and BCPAP cells which were co-transfected with TOP-Flash and LINC01278 siRNA (LINC-KD) or overexpression plasmid (LINC01278). **C** The enrichment of LINC01278 in β-catenin protein by using RNA immunoprecipitation and qRT-PCR. ACTB was used as the control. **D** LINC01278 precipitated β-catenin in TPC-1 and BCPAP cells detected by RNA pulldown and western blot. *P < 0.05

**Fig. 5:**
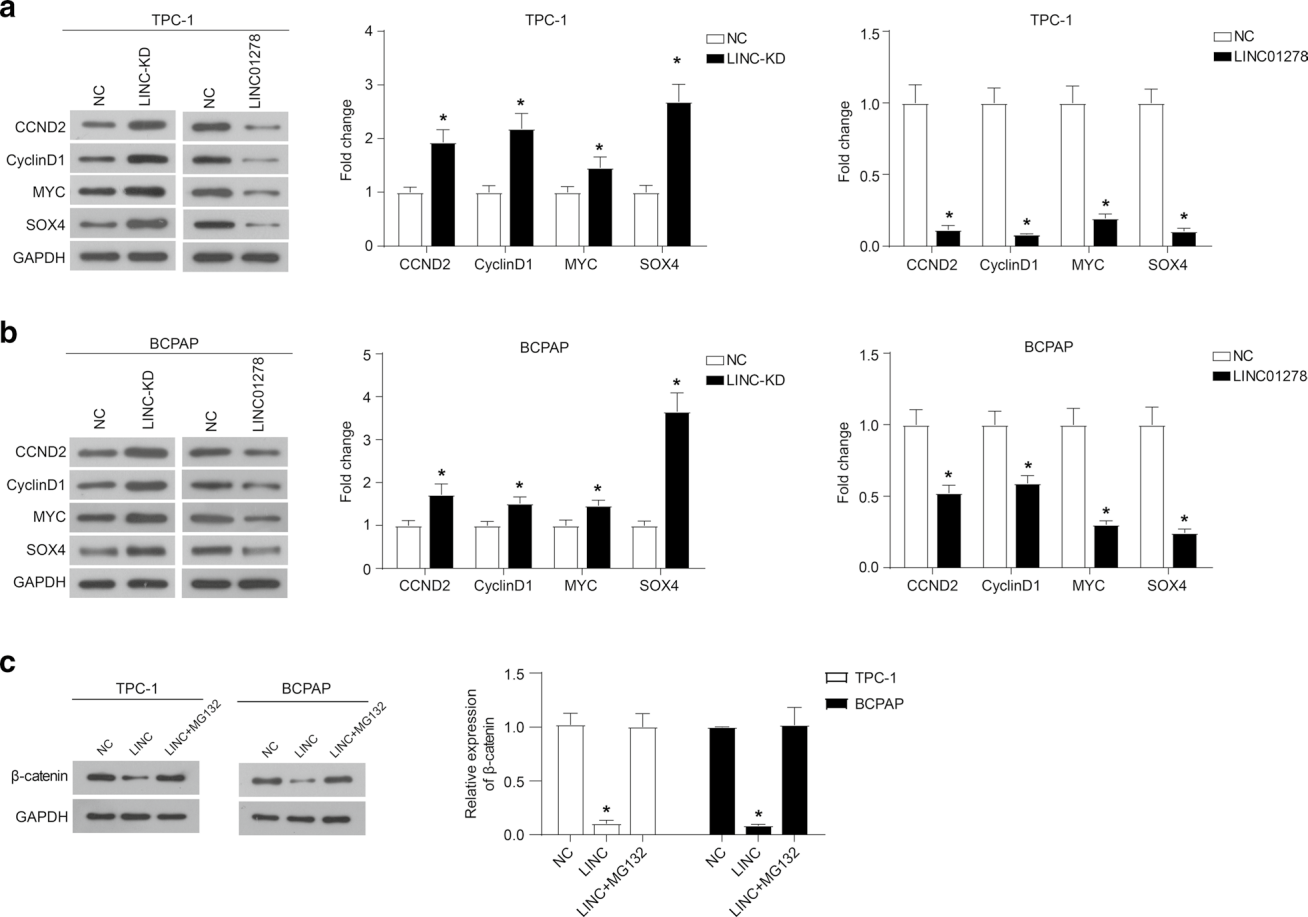
LINC01278 promotes the ubiquitin–proteasome degradation of β-catenin. The expression of Wnt/β-catenin signal targets (CCND2, CyclinD1, MYC, and SOX4) in TPC-1 (**A**) and BCPAP (**B**) cells which were transfected with LINC01278 siRNA (LINC-KD) or overexpression plasmid (LINC01278). **C** The protein expression of β-catenin in TPC-1 and BCPAP cells which were treated with a proteasome inhibitor (MG132) and LINC01278 expression plasmid. *P < 0.05

Our previous research indicated that LINC01278 inhibited the epithelial-to- mesenchymal transition (EMT) of TPC-1 and BCPAP cells [11]. It is well known that the activation of the Wnt/β-catenin signal pathway regulates the EMT[16]. Therefore, we speculated that Wnt/β-catenin signal activates the transcription of LINC01278, and the accumulation of LINC01278 could negatively regulate the activation of Wnt/β-catenin signal. As shown in Fig. 4A, the down-regulation of LINC01278 expression increased the accumulation of β-catenin in the cytoplasm and nucleus. In addition, the up-regulation of LINC01278 expression reduced the level of β-catenin protein in the cytoplasm and nucleus. Furthermore, the results of TOP/FOP luciferase experiments showed that knocking down LINC01278 promoted the luciferase activity, and overexpression of LINC01278 inhibited the luciferase activity (Fig. 4B). The results of RNA immunoprecipitation and qRT-PCR indicated the enrichment of LINC01278 on β-catenin protein (Fig. 4C). Then, RNA pulldown and western blot also confirmed the mutual binding of LINC01278 and β-catenin (Fig. 4D). In addition, the knockdown of LINC01278 significantly increased the expression of Wnt/β-catenin signaling pathway targets (CCND2, CyclinD1, MYC, and SOX4), while the overexpression of LINC01278 suppressed the expression of these proteins (Fig. 5A and B). These data indicated the interaction of LINC01278 withβ-catenin.

### LINC01278 promotes the ubiquitin–proteasome degradation of β-catenin

In order to study how LINC01278 regulates the protein level of β-catenin, we added a proteasome inhibitor (MG132) to TPC-1 and BCPAP cells overexpressed by LINC01278, and found that MG132 relieved the effect of LINC01278 overexpression on β-catenin protein levels (Fig. 5C). These results indicated that the combination of LINC01278 and β-catenin promoted the ubiquitin–proteasome degradation of β-catenin.

## Discussion

**Fig. 6:**
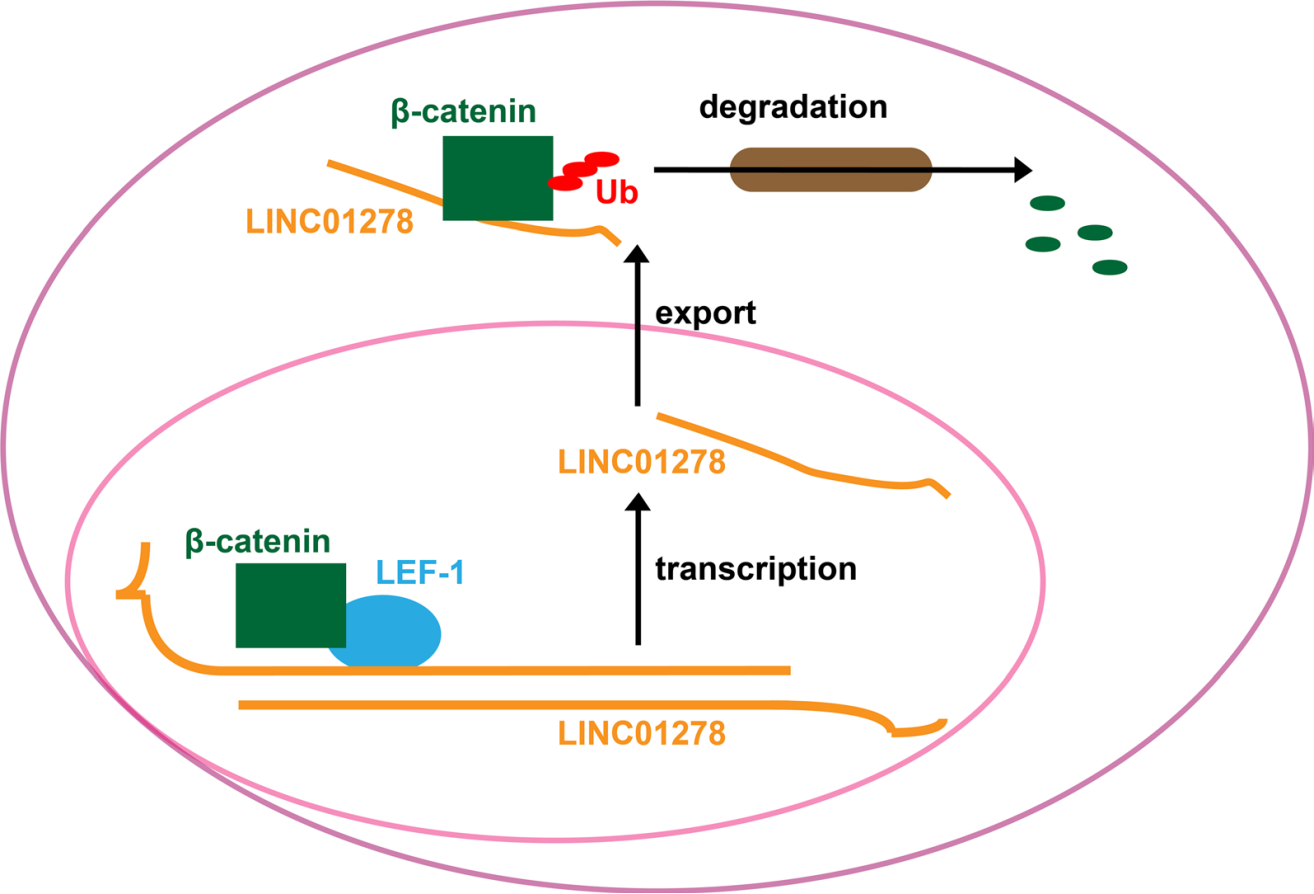
Molecular mechanism schematic diagram

In recent years, the important role of lncRNAs has continued to appear in a variety of physiological and pathological processes, including tumorigenesis. Our previous research found that LINC01278 was significantly down-regulated in PTC tissues and cell lines, and exerted a tumor suppressor function in tumor cells [11]. However, the study by Huang et al. reports that LINC01278 promotes the metastasis of hepatocellular carcinoma (HCC) by targeting miR-1258-Smad2/3 [17]. In addition, Qi et al. find that the expression of LINC01278 in osteosarcoma tissues is enhanced, is the increased linc01278 expression is related to clinical staging, distant metastasis and poor prognosis of patients, and LINC01278 promotes the proliferation of osteosarcoma cells through miR-133a-3p/PTHR1 signal [18]. These two latest studies suggest that LINC01278 plays a role of promoting tumor progression in HCC and osteosarcoma, which is contrary to the tumor suppressor effect of LINC01278 in thyroid cancer. The role of LINC01278 in tumor cells may depend on the specific tumor types, tumor microenvironment or downstream targets. In this study, we revealed the transcriptional activation of LINC01278 by the β-catenin/ LEF-1 transcription factor and the negative feedback regulation of LINC01278 on the degradation of β-catenin (Fig. 6).

We cloned the LINC01278 promoter sequence containing the wild-type or mutant-type putative LEF-1 binding site into the firefly luciferase reporter system vector. The combination of transcription factor LEF-1 and promoter site can regulate the expression of downstream firefly luciferase. This experiment confirmed the binding of transcription factor LEF-1 to the promoter site of LINC01278. ChIP experiments also confirmed the endogenous binding of the transcription factor LEF-1 to the LINC01278 promoter, and showed that β-catenin can enhance the binding of LEF-1 to the LINC01278 promoter. The results of western blot showed that knockdown or overexpression of LEF-1 or β-catenin and specific agonists or inhibitors of the Wnt/β-catenin pathway can affect the expression level of LINC01278. Our results discovered for the first time the regulatory mechanism of Wnt/β-catenin/LEF-1 signaling on the transcriptional activation of LINC01278.

In addition, we discovered the negative feedback regulation of LINC01278 on β-catenin signal. Western blot and TOP/FOP luciferase experiments suggested that LINC01278 negatively regulated the protein accumulation of β-catenin in the cytoplasm, into nucleus, and ultimately inhibited the transcriptional of downstream target genes activated by Wnt/β-catenin signals. The results of RNA IP and RNA pulldown proved the direct binding of LINC01278 to β-catenin. Subsequently, the application of proteasome inhibitors suggested that the combination of LINC01278 and β-catenin can promote β-catenin ubiquitination-proteasome degradation. It is currently known that β-catenin has a variety of regulatory mechanisms, such as APC degradation complex and CTNNBIP1. Here, we reported the regulatory mechanism of a new lncRNA on β-catenin signal. In addition, the relationship between LINC01278 and other Wnt/β-catenin signal regulators needs to be further study.

## Conclusion

In conclusion, we found the transcriptional activation of LINC01278 by the β-catenin/LEF-1 transcription factor, and the negative feedback regulation of LINC01278 on β-catenin signal. This is the first report on the regulation mechanism of LINC01278 expression, as well as a new mechanism report on the β-catenin signal activation. Although, the specific interaction between them needs more research.

## Data Availability

Not applicable.
